# Detection of multimycotoxins in camel feed and milk samples and their comparison with the levels in cow milk

**DOI:** 10.1002/fsn3.2677

**Published:** 2021-12-13

**Authors:** Randa Zeidan, Zahoor Ul Hassan, Noor Al‐Naimi, Roda Al‐Thani, Samir Jaoua

**Affiliations:** ^1^ Environmental Science Program Department of Biological and Environmental Sciences College of Arts and Sciences Qatar University Doha Qatar; ^2^ Anti‐Doping Lab Qatar Doha Qatar

**Keywords:** camel and cow milk, camel feed, food safety, mycotoxins, synergistic toxicity

## Abstract

Camel milk has been considered as an important source of nutrients and is commercialized in many countries of the world including the Middle East. This study aimed to investigate the presence of mycotoxins in camel feed and milk samples in comparison with the cow milk. Fumonisins (FUM), ochratoxin A (OTA), and zearalenone (ZEN) were detected in 14%, 39%, and 39% of the tested camel feed samples, respectively. Among the tested camel feed samples, 8.3% and 5.6% were co‐contaminated with OTA+FUM and FUM+ZEN, respectively. In the case of milk samples, 46.15% of camel and 63.63% of cow were found contaminated with aflatoxin M1 (AFM1). In total, 16.2% and 8.1% of the milk samples were simultaneously contaminated with two and three mycotoxins, respectively. Although the levels of individual mycotoxins in the camel feed and milk samples were within the European Union (EU) permissible limits, their co‐occurrence may pose severe risk to human and animal health due to possible additive and/or synergistic toxicities.

## Graphical abstract

Fumonisins, ochratoxin A, and zearalenone were detected in camel feed samples. Among the tested camel feed samples, some were co‐contaminated with OTA+FUM and FUM+ZEN. In the case of milk samples, 46.15% of camel and 63.63% of cow were found contaminated with aflatoxin M1 (AFM1). Although the levels of individual mycotoxins in feed and milk samples were within the European Union (EU) permissible limits, their co‐occurrence may pose severe risk to human and animal health due to possible additive and/or synergistic toxicities.

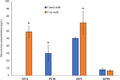

## 1 INTRODUCTION

Camel is a domesticated animal, which is an important resource of food in many countries of the world including the Middle East. The health benefits of camel milk are associated with its antimicrobial (Abdel‐Hamid et al., 2016), dislipedemic (Mohammaddin et al., 2018), antidiabetic (Fallah et al., 2020), and anticancer (Abrhaley & Leta, 2018) properties, and its richness of minerals and vitamins (Swelum et al., 2021).

Mycotoxins, the metabolites of some fungal spp., are significant environmental contaminants in agricultural products, especially in the cereals and grains. The important toxigenic fungi, which infect the agricultural crops or their products at different production and storage stages, include members of the genus *Aspergillus*, *Penicillium*, or *Fusarium* (Hassan et al., 2018,2019). Like other animals, camel's diet consists of cereals, grains, and dried or green roughages. The natural occurrence of toxigenic fungi and their mycotoxins in camel feed are frequently observed (Almoammar et al., 2014; Bokhari, 2010). In countries such as Qatar, the camel feed ingredients are imported from the regions of diverse climatic conditions; therefore, the feed contamination with a variety of toxigenic fungi is prevalent. These feed ingredients are usually mixed to formulate complete rations leading to the co‐occurance of a wide range of mycotoxins (Hassan et al., 2019; Hassan, Al‐Thani, Migheli, et al., 2018). Apart from the harmful effects of dietary mycotoxins on the animals health, the residual transfer through animal products (meat and milk) into human food chain poses a risk to human health. The occurrence of multimycotoxins, even if the level of each individual compound is within the permissible limits, is an emerging issue due to recent findings on their synergistic or additive toxicities (Kifer et al., 2020; Sobral et al., 2018). Restricted nutrition intake puts certain groups at risk of exposure to mycotoxins and develops other diseases (Valitutti et al., 2017,2018).

Aflatoxin M1 (AFM1), a hepatic metabolite of aflatoxin B1 (AFB1), is a frequently detected mycotoxin in animal milk and other body secretions (Flores‐Flores et al., 2015; Hassan, Al‐Thani, Atia, et al., 2018; Min et al., 2021). Like the parent toxin (AFB1), AFM1 is also a known carcinogen (toxicity is 10 times lower than AFB1) and is the only regulated mycotoxin in the milk. Many countries around the world follow the European Union (EU) permissible limit of 50 ng/L in milk, while the Food and Drug Administration has 10 times higher permissible limit of 500 ng/L (European Commission, 2006; FDA, 2020). To date, food regulatory authorities around the globe have not set permissible limits for several mycotoxins in milk, including ochratoxin A (OTA) and the toxins of *Fusarium* spp. such as zearalenone (ZEN), fumonisin (FUM), and deoxynivalenol (DON).

In dairy animals such as cows, there are studies concerning the gut microbe degradation and biotransformation of mycotoxins by the liver microsomal enzymes, and such processes lead to a lower release of mycotoxins (1%–6% of the total dietary exposure) in milk (Fink‐Gremmels, 2008; Min et al., 2021). However, little is known about the feed‐to‐milk carryover of mycotoxins in camel.

Keeping up with the aforementioned knowledge gaps, this study has been designed to investigate the levels and types of mycotoxins in camel feed and in milk collected from the feed market and camel farms in Qatar. In parallel, milk samples from cows were also analyzed for the presence of multimycotoxins for the purpose of comparing their levels in these two important dairy animals.

## 2 MATERIAL AND METHODS

### 2.1 Sampling

In this study, a total of 36 feed and 37 milk samples were collected from the feed market and camel farms located in Qatar. All the samples were packed separately in sterile airtight bags and transported to the Department of Biological and Environmental Sciences, Qatar University. Based on their nature, the camel feed samples were divided into cereal‐/grain‐mixed (*n* = 23), dry fodder (*n* = 08), and green fodder (*n* = 05). Milk samples were separated into camel milk (*n* = 26) and cow milk (*n* = 11). Before analysis, feed samples were ground to powder using a blender (for grains and dry fodder) or pestle and mortar in the presence of liquid nitrogen (for green fodder). All samples were preserved in 50‐ml tubes at 4°C in fridge prior to the mycotoxins extraction.

### 2.2 Extraction and analysis of mycotoxins from the camel feed samples

#### 2.2.1 Extraction and analysis of ochratoxin A (OTA)

All the feed samples were extracted and analyzed for the presence of OTA by following the instructions described in ELISA kit (RIDASCREEN^®^ Ochratoxin A 30/15; R‐Biopharm AG). Of the ground feed samples, 2 g was suspended in 5 ml of 1N HCl and was mixed for 5 min. To each tube, 10 ml of dichloromethane was added, and the samples were left for 15 min in ashaker. After centrifugation, the upper phase was removed and the rest of the tube contents were filtered using the Whatman filter paper. To the filtrate, equal volume of 0.13 M of NaHCO_3_ was added. After a thorough mixing for 15 min, tubes were centrifuged again. A total volume of 100 µl from the upper phase was diluted in 400 µl of sodium hydrogen carbonate (0.13 M). To the duplicate ELISA wells, 50 µl of the diluted filtrate was added. Microplate reader (Tecan Sunrise™) was used to measure the absorbances at 450 nm. Data were acquired using Tecan Magellan software, and mycotoxin concentrations in the samples were obtained on the basis of calibration curve using RIDA^®^Soft Win‐Z9996 (R‐Biopharam).

#### 2.2.2 Extraction and analysis of fumonisins (FUM) and zearalenone (ZEN)

The extraction of FUM and ZEN from camel feed was carried out by following the protocol described in ELISA kits, RIDASCREEN^®^ Fumonisin (R3401) and RIDASCREEN^®^ Zearalenone (R1401), respectively. Briefly, 5 g of ground feed samples was mixed with 25 ml of 70:30 methanol:water and was shaken and incubated for 3 min. The tubes were centrifuged, and the supernatant was diluted in 1.3‐ml dH_2_O for FUM extraction. For ZEN extraction, the supernatant was diluted (1:7) with the buffer provided with the ELISA kit. In both cases, 50 µl of the diluted samples was applied in the ELISA wells. Absorbance and mycotoxin concentrations were calculated as described in section 2.2.1 above.

### 2.3 Mycotoxin analysis in milk

All milk samples were skimmed before analysis for the presence of mycotoxins. For this purpose, 5 ml of milk was centrifuged at 3500 × *g* for 10 min. The layer of fat was scraped off, and fat‐free samples were shifted to new tubes. Levels of aflatoxin M1 (AFM1), OTA, ZEN, and FUM were determined using ELISA kits as described above. In each case, 50 µl of the skimmed milk samples was applied to the duplicate wells of the respective ELISA plates. Absorbance and mycotoxin concentrations were calculated as described above (section 2.2.1) using the microplate ELISA reader and RidaWin^®^ software, respectively.

### 2.4 Statistical analysis

The data on the prevalence of mycotoxins in camel feed and milk samples were presented in percentage (%) of the positive samples detected. Further comparisons were made on the basis of the feed nature and sample source. Levels of different mycotoxins in camel feed and milk samples were presented in ranges (minimum‐maximum) and their mean values. Analysis of variance (ANOVA) was performed for the comparison between mycotoxins levels in the camel and cow milk samples. SPSS software was used to analyze the data.

## 3 RESULTS AND DISCUSSION

### 3.1 Mycotoxins in camel feed

#### 3.1.1 Prevalence of mycotoxins in different types of camel feed

**FIGURE 1:**
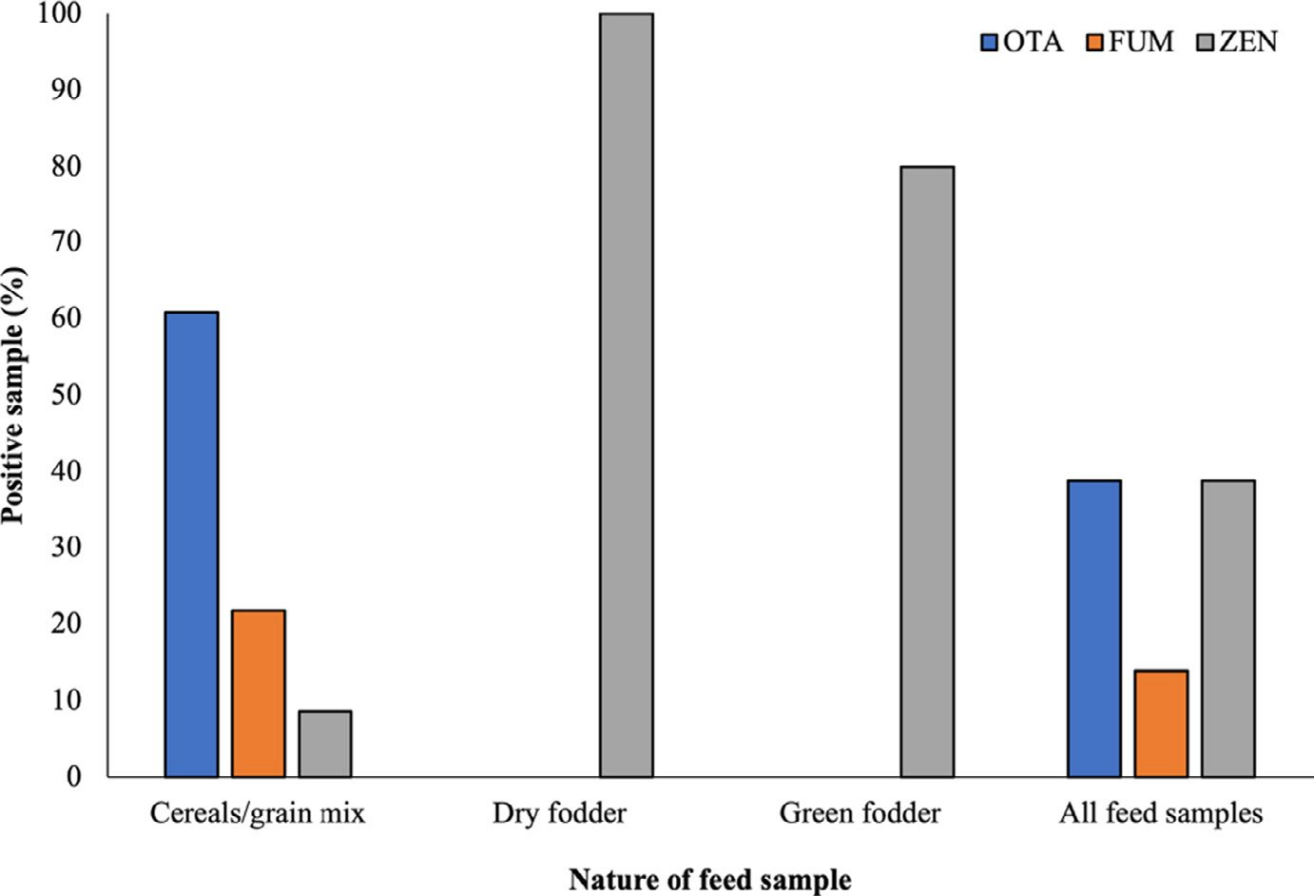
Mycotoxins in camel feed (percentage of positive samples) according to the nature of the samples. OTA and FUM were detected in the cereal‐/grain‐mixed feed only, while ZEN was found in all three types of feed. A significantly higher percentage of the dry and green forage samples were contaminated with ZEN, compared with cereals and grains

Mycotoxin‐associated pathologic outcomes and altered performance in camels are poorly studied; however, like other animals, camel is also susceptible to mycotoxicosis as reported in the natural and experimental exposure in UAE (Osman et al., 2004) and Saudi Arabia (Al‐Hizab et al., 2015). In the present study, a total of 36 camel feed samples were collected from the camel feed market and camel farms located in Qatar and analyzed for the occurrence of ochratoxin A (OTA), fumonisins (FUM), and zearalenone (ZEN). Among the cereal‐/grain‐mixed feed, FUM and OTA were detected in 60.86% and 21.73% of the samples, respectively (Figure 1). In Saudi Arabia, for testing camel feed, Bokhari (2010) found that 85% of total 40 samples were positive for the OTA contamination. Comparatively, lower OTA contamination in this study might be associated with the nature of samples. In the present investigation, OTA was not detected in green and dry fodder samples. This is presumably due to lesser susceptibility of fodder to ochratoxigenic *Aspergillus* and *Penicillium* infection compared with the cereals and grains. ZEN, an estrogenic metabolite of *Fusarium* spp., was detected in 100%, 80%, and 8.6% of the dry fodder, green grasses, and cereal‐/grain‐mixed feed samples, respectively. The presence of ZEN in animal feed is frequently reported at different significant levels between grains and forges. In one of our previous studies, in the marketed feed grains in Qatar, ZEN was detected in 40% (wheat) to 85.5% (mixed grains) of the samples (Hassan et al., 2019). Apart from the other factors, seasonal variations, presence of competitor micro‐organisms, toxigenic potential of the infecting fungal strains, nature of the substrates, and origin of the samples (import country) play an important role in the mycotoxin accumulation. Overall, 38.9%, 13.9%, and 38.9% of the total tested feed samples were positive for OTA, FUM, and ZEN, respectively (Figure 1).

#### 3.1.2 Effect of feed samples source on the occurrence of mycotoxins

**FIGURE 2:**
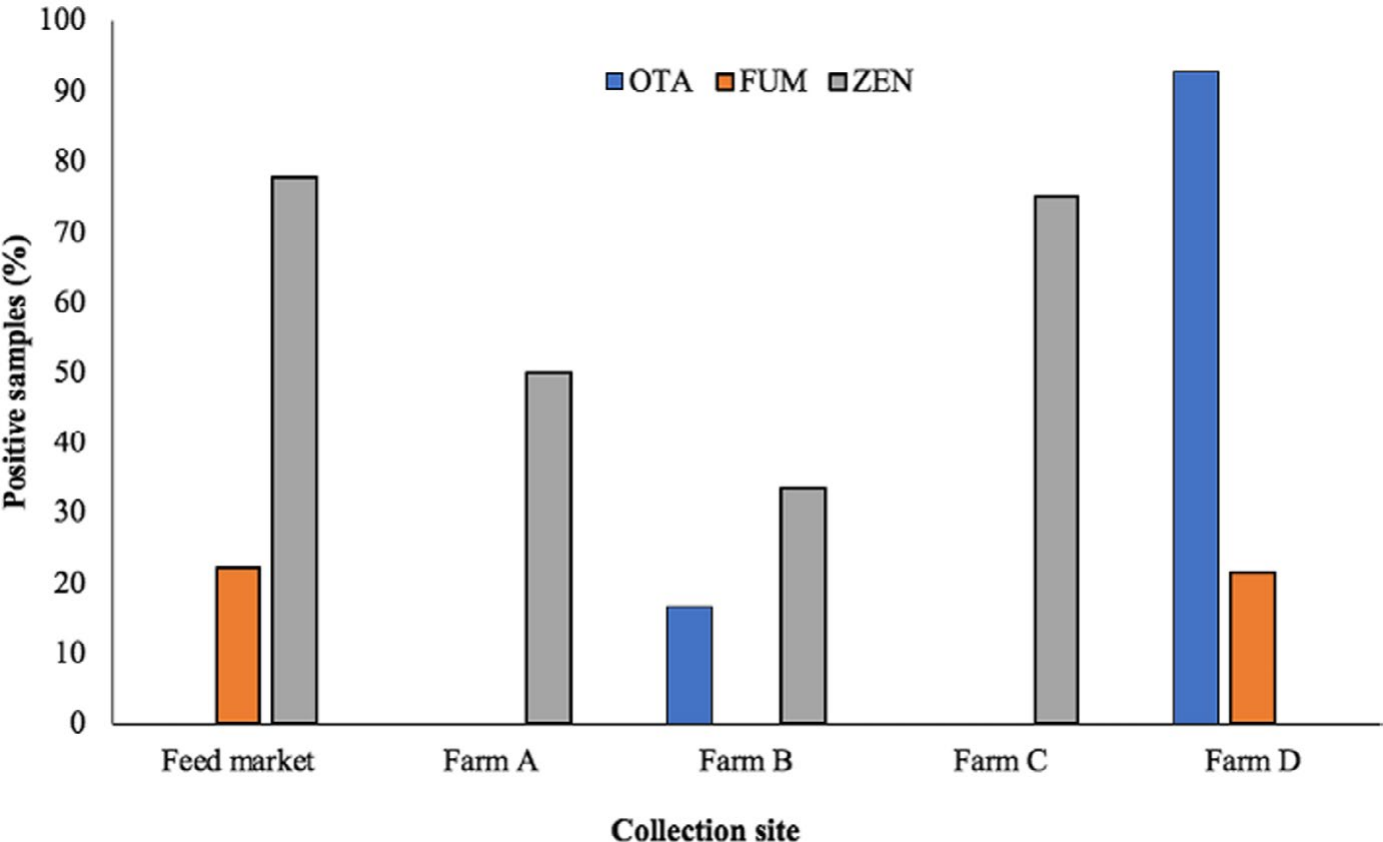
Mycotoxins (percentage of positive samples) in camel feed collected from different sites. ZEN was detected in all the sites where forages samples were collected. OTA and FUM were specifically found in the grain/cereal sample sites

In order to explore the possible effect of storage of imported feed samples on the mycotoxin levels, samples were collected from the camel feed market and from the camel farms. ZEN was the most prevalent mycotoxin and was found in the samples collected from the feed market and from all camel farms, except for farm D (all its samples were cereal‐based). The presence of ZEN showed an association with the nature of feed, being present more in the dry and green grass samples compared with the grains (Figure 2). OTA was not detected in the samples collected from the feed market and neither from farm A nor from farm C. In the feed market, feed bags are generally kept for shorter time before being transported to the animal farms. The absence of OTA in feed market samples is possibly associated with the shorter storage duration as compared to the samples collected from farms B and D. OTA contamination data on the samples from farm D (all samples were grain/cereal‐based) strengthen this hypothesis, where 92.85% of the samples were positive for OTA contamination. The absence of OTA in farms A and C is likely associated with the nature of feed as none of the collected samples from these locations were cereal or gain based. These findings are augmented by the fact that *Aspergillus* and *Penicillium* are generally referred to as storage fungi compared with *Fusarium* spp., which infect the crops in field conditions and hence are referred to as field fungi. In line with our findings, Skládanka et al. (2011) found only *Fusarium* mycotoxins ZEN and DON during the growing season of grass forage, while no *Aspergillus* mycotoxins (aflatoxins) were found. Likewise, in our findings, ZEN also was not found in the growing forage samples.

#### 3.1.3 Level of mycotoxins in camel feed in relation to EU permissible limits

There are no specific regulatory limits for mycotoxins in camel feed. Considering the EU limits in feedstuff for other dairy animals, in this study, the levels of all detected mycotoxins were within the maximum permissible limits. OTA was detected only in the cereal‐ or grain‐mixed samples with levels ranging from 0.23 to 9.44 ng/g (Table 1). These levels are far below 250 ng/g, which is the EU permissible limit for OTA in feedstuff (European Commission, 2002). Furthermore, the levels of FUM (4.16–12.37 µg/g) and ZEN (1.92–22.80 ng/g) in the camel feed samples were also much lower than the EU permissible limits, which are 50 mg/kg and 2 mg/kg, respectively, for other animals (European Commission, 2006). In line with the framework of this study, Almoammar et al. (2014) reported the levels of mycotoxins (aflatoxins) in camel feed in the range of 1–3.2 µg/kg compared with the EU set level of 20 µg/kg in animal feed.

**TABLE 1:** Levels of mycotoxins in the camel feed samples

Nature of feed	OTA^a^ Range (mean)	FUM^b^ Range (mean)	ZEN^a^ Range (mean)
Cereals/grain mix	0.23–9.44 (1.98)	4.16–12.37 (7.41)	1.92–1.92 (1.92)
Dry fodder	nd^c^	nd	2.01–22.8 (8.72)
Green fodder	nd	nd	2.50–9.60 (5.36)
All feed samples	0.23–9.44 (1.98)	4.16–12.37 (7.41)	1.92–22.80 (6.79)

Note

All the camel feed samples were contaminated with OTA, ZEN, and FUM at levels within the EU permissible limits.

^a^ Levels of mycotoxins are expressed in ng/g;

^b^ Levels of mycotoxin are in µg/g;

^c^ Below the limit of detection.

### 3.2 Mycotoxins in the camel and cow milk samples

**FIGURE 3:**
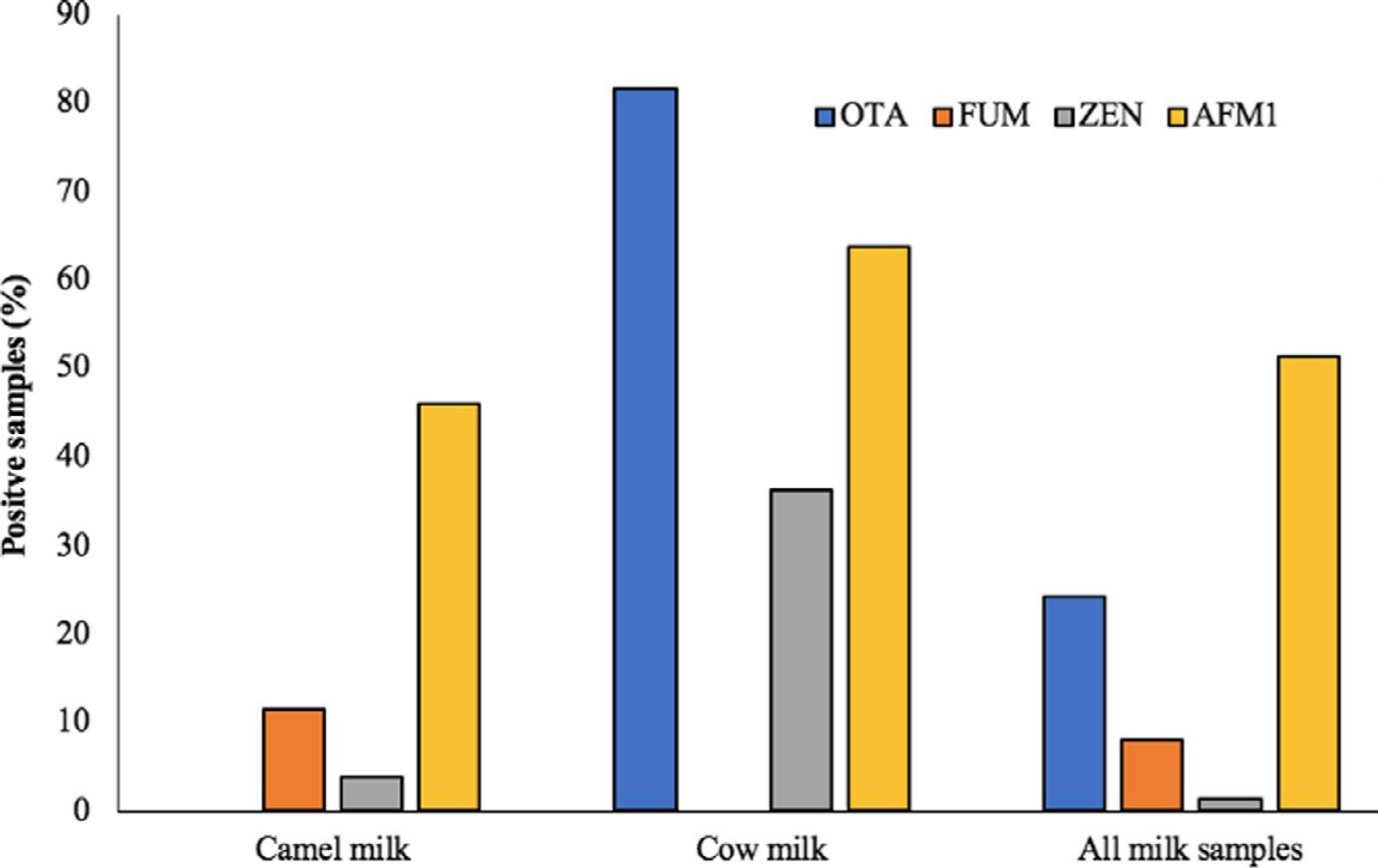
Mycotoxins in camel and cow milk (percentage of positive sample). OTA was detected in the cow milk, while FUM was found in camel milk only. A significant percentage of the camel and cow milk samples were contaminated with AFM1

Milk is rarely tested for the contamination with mycotoxins other than AFM1. In this work, a significant number of camel (46.15%) and cow milk samples (63.63%) were tested positive for AFM1 contamination (Figure 3). Yousof and Zubeir (2020) in Sudan reported even lower AFM1 contamination in camel milk (15.6%) compared with a higher (82.2%) occurrence in the cow milk. The lower incidence of AFM1 in camel milk compared with the cow may be associated with (a) lower dietary intake of parent AFB1, as camels are offered less feed concentrate compared with bovine feeding regimes; (b) activity of camel's ruminal microflora that leads to more degradation of AFB1; (c) intestinal morphological differences impeding the absorption of AFB1 in camel; or (d) activity of hepatic microsomal enzymes leading to the biodegradation of AFB1 to other biotransformed metabolites different than AFM1 in camel. In the detailed study, all these hypotheses can be studied one by one to arrive at precise conclusion.

Among the cow milk samples, 81.8% were found contaminated with OTA, which was not detected in any of the camel milk samples. Relatively less percentage of the camel milk samples were contaminated with FUM and ZEN at 11.53% and 3.84%, respectively. On the contrary, FUM was not detected in the cow milk samples, while ZEN was found comparatively higher (36%) in the samples. The presence of FUM in milk has been reported earlier by Maragos and Richard (1994) in 0.6% of the tested samples and by Gazzotti et al. (2009) in 80% of the samples. Likewise, the occurrence of ZEN in raw cow milk has been reported by El‐Hoshy (1999) in 20% of the tested samples. Overall, a higher percentage of the cow milk samples were contaminated with mycotoxins compared with the camel milk.

**FIGURE 4:**
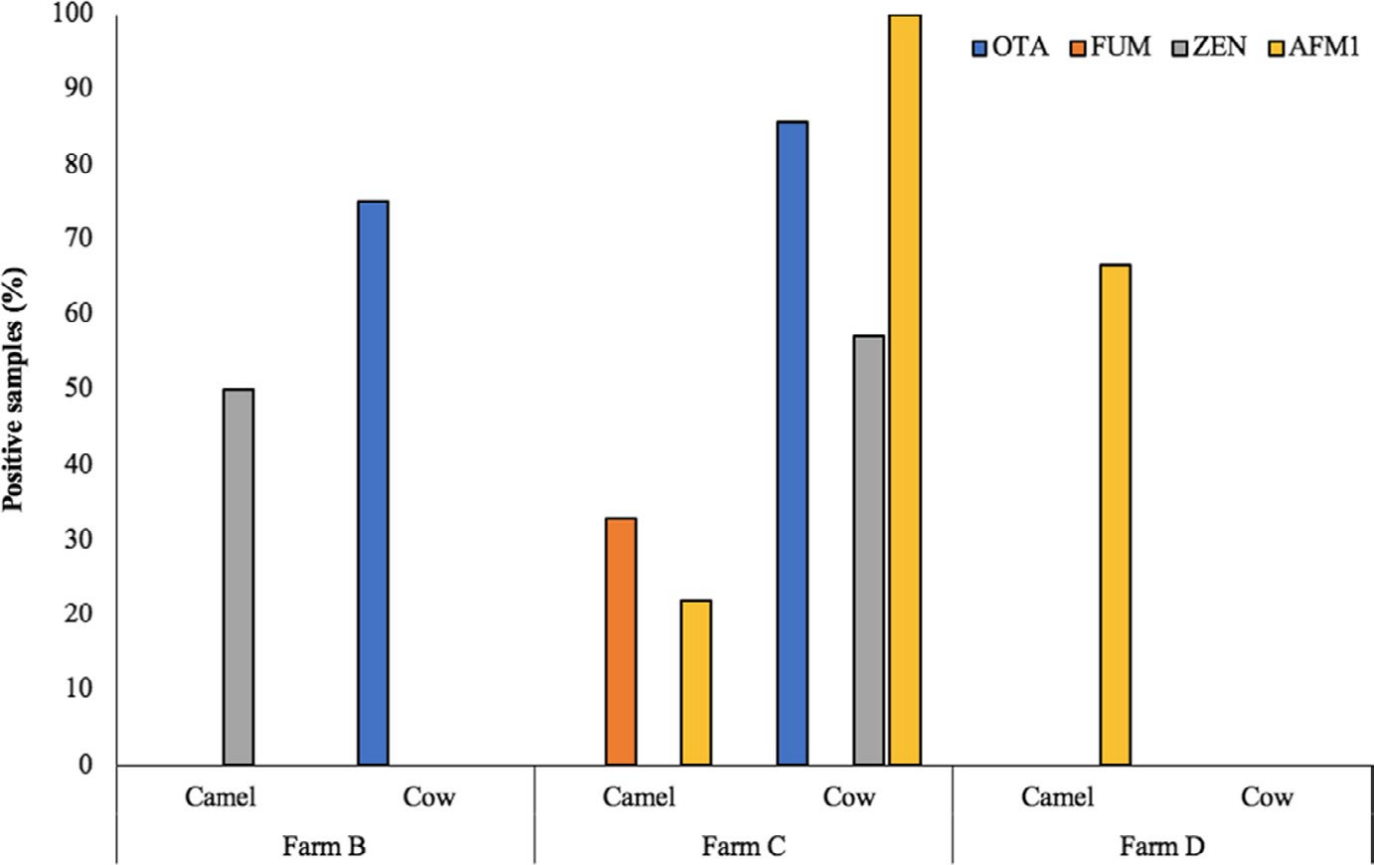
Farm‐wise comparison of the camel and cow milk samples for mycotoxins contamination (percentage of positive samples). AFM1 and OTA were the most frequently detected mycotoxins in the milk samples collected from different farms

AFM1 was detected neither in the camel milk nor in the cow milk samples from farm B (Figure 4). However, 50% of the camel milk samples were contaminated with ZEN, and 75% of the cow milk samples were positive for OTA. In farm C, OTA and ZEN were not found, while FUM and AFM1 were found in 33% and 22% of the tested samples, respectively. In farm C, AFM1, OTA, and ZEN were detected in 100%, 85.71%, and 57.14% of the cow milk samples, respectively. In farm D, 66.7% of the samples obtained from camel were positive for AFM1 contamination.

**FIGURE 5:**
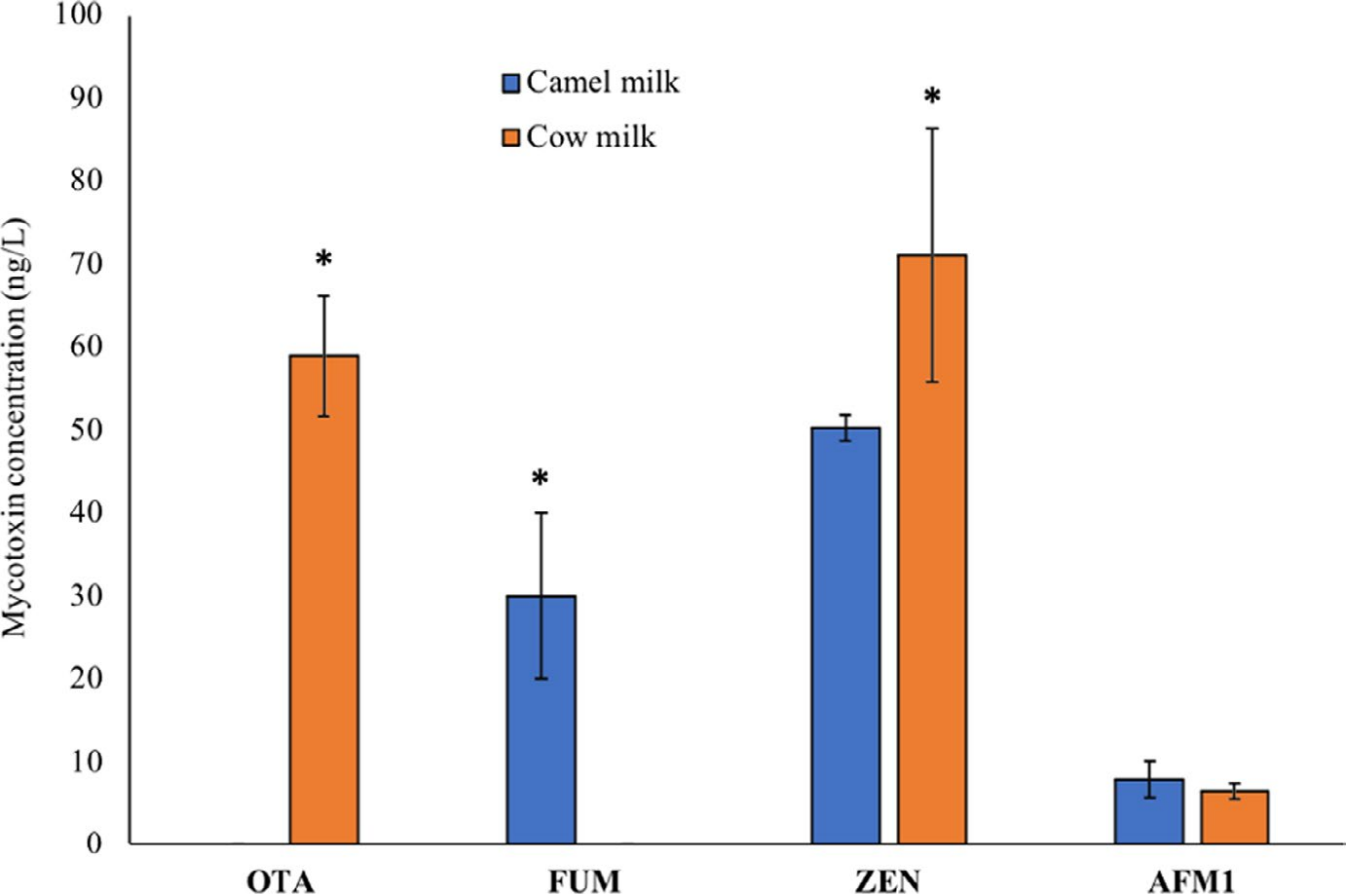
Levels of mycotoxins (ng/L) in camel versus cow milk. Levels of AFM1 in camel and cow milk were nonsignificant from each other. Levels of ZEN and OTA in cow milk were significantly higher than that in camel milk

OTA was not detected in any of the camel milk samples, while FUM, ZEN, and AFM1 were found in the ranges of 28–38 ng/L, 50.31 µg/L, and 5.32–12.73 ng/L, respectively (Figure 5). In accordance with the study outcomes, the occurrence of OTA in the cow milk has been previously reported in France (Boudra et al., 2007), Italy (Pattono et al., 2011), and China (Huang et al., 2014). The levels reported in Italy (70–110 ng/L) were higher, while those in France (5–6.6 ng/L) were much lower than those observed in our study. The detected levels of AFM1 in camel and cow milk in the current results were not significantly different from each other. Nevertheless, according to the previous studies such as that conducted by Omar (2016), he reported significantly lower AFM1 levels in camel milk (37.15 ng/kg) compared with the marketed cow (68.91 ng/kg), sheep (70.25 ng/kg), and goat (60.25 ng/kg) milk in Jordan. In Abu Dhabi, Saad et al. (1989) found AFM1 in 30% of the tested camel milk samples in the range of 0.25–0.8 ng/ml, which were far below the levels detected in the present study. Throughout the world, none of the mycotoxins has a set regulation in milk except for AFM1. In this study, the levels of AFM1 in camel milk and in cow milk were much lower than the EU permissible limit of 50 ng/L. The levels of FUM (28–38 µg/L) in camel milk observed in the present study were much lower than what was reported in studies about cow milk by Maragos and Richard (1994) (1290 µg/L) and Gazzotti et al. (2009) (260–430 µg/L). In this research study, the levels of ZEN in camel milk were significantly lower than those in the cow milk samples. In China, the reported levels of ZEN (14.9–45.8 ng/L) by Huang et al. (2014) in raw cow milk were lower than that in the levels found in our study. However, the Scientific Cooperation on Questions Relating to Food (SCOOP, 2003) reported much higher levels (500–5500 ng/L) of ZEN in the United Kingdom.

### 3.3 Occurrence of multimycotoxins in feed and milk samples

The levels of mycotoxins in food are regulated based on their occurrence data and toxicological implications as individual toxins. On the contrary, agricultural crops and feed grains are generally contaminated with more than one toxigenic fungus, leading to the accumulation of multimycotoxins in the matrix. In countries such as Qatar, cereal‐/grain‐mixed rations are prepared by mixing the ingredients imported from different geographical regions having different fungal profiles, and this leads to the co‐contamination with multimycotoxins in fodder and animal products. In such cases, although the levels of individual mycotoxins are within the permissible ranges, their synergistic or additive toxic impacts create health risk for the exposed subjects (Queiroz et al., 2012; Smith et al., 2016). There are studies where the co‐exposure to more than one mycotoxin was proven to result in significant toxicological outcomes (Sobral et al., 2018; Sun et al., 2014).

In this work, among all the tested camel feed samples, 41.1% were positive for the contamination of either OTA, ZEN, or AFM1 alone, while 8.3% and 5.6% of the samples were concurrently contaminated with OTA+FUM and FUM+ZEN, respectively (Table 2). In case of the tested milk samples (camel and cows), 40.5% were contaminated with either OTA, ZEN, FUM, or AFM1. In total, 16.2% of the samples were contaminated with two mycotoxins and 8.1% of milk samples were concurrently contaminated with three mycotoxins (OTA+ZEN+AFM1). In accordance with our findings, Huang et al. (2014) in China detected multiple mycotoxins in single milk sample, with 22% being contaminated with 4 mycotoxins, 45% with 3 mycotoxins, and 15% with 2 mycotoxins. It is worth mentioning that in this study, the uniqueness of the analysis relies on the fact that the samples of milk were collected from each animal individually (camel or cow). Hence, each sample is remarkable and represents a singular animal, as this type of sampling is usually challenging to attain.

**TABLE 2:** Co‐occurrence, incidence, and frequency of mycotoxins in camel feed and milk samples

*N*° mycotoxins	Co‐occurrence of mycotoxins	Incidence	Frequency
**Feed (*n* = 36)**			
1	OTA	1	2.7%
	ZEN	13	36.1%
	FUM	1	2.7%
**Total**		**15**	**41.7%**
2	OTA, FUM	3	8.3%
	FUM, ZEN	2	5.6%
**Total**		**5**	**13.9%**
**Milk (*n* = 37)**			
1	OTA	3	8.1%
	ZEN	1	2.7%
	FUM	1	2.7%
	AFM1	10	27.0%
**Total**		**15**	**40.5%**
2	FUM, AFM1	2	5.4%
	OTA, AFM1	3	8.1%
	ZEN, AFM1	1	2.7%
**Total**		**6**	**16.2**%
3	OTA, ZEN, AFM1	3	**8.1%**

Note

Bold indicates total feed sample basis, 41.7% and 13.9% were contaminated with one and two mycotoxins, respectively. Out of the total tested milk samples, 16.2% were contaminated with two and 8.1% with three mycotoxins simultaneously.

## 4 CONCLUSION

A significant number of camel feed samples imported to Qatar are contaminated with zearalenone (ZEN), fumonisins (FUM), and ochratoxin A (OTA). The nature of the mycotoxins in feed is associated with the type of feed, with cereals or grains having more OTA than green and dry forages. Camel feeds are often contaminated with more than one mycotoxin, such as OTA with FUM and FUM with ZEN. A significant number of milk samples (camel and cow) are contaminated with AFM1, along with FUM, ZEN, and OTA. Although the levels of individual mycotoxins in feed and milk were within the maximum permissible limits set by EU, still their co‐occurrence may pose severe risks to human and animal health due to possible additive or synergistic toxic effects.

## CONFLICT OF INTEREST

The authors declare that there is no conflict of interest in this research.

## AUTHOR CONTRIBUTIONS

**Randa Zeidan** conceptualization (supporting) ; methodology (lead); validation (equal);writingOriginalDraft (supporting); writingReviewEditing (supporting). **Zahoor Ul Hassan** conceptualization (supporting) ; methodology (supporting); validation (supporting); writingOriginalDraft (supporting);writingReviewEditing (supporting). **Noor Al‐Naimi** methodology (supporting) . **Samir Jaoua** conceptualization (lead) ; formalAnalysis (supporting); fundingAcquisition (lead);investigation (lead); methodology (supporting);projectAdministration (lead); resources (lead)software(lead);supervision (lead); validation (supporting); writingOriginalDraft(supporting);writingReviewEditing (lead).

## ETHICAL APPROVAL

Ethics approval was not required for this research.

## Data Availability

Data available on request due to privacy/ethical restrictions.
